# A Case-Control Study and Meta-Analysis Reveal* BDNF* Val66Met Is a Possible Risk Factor for PTSD

**DOI:** 10.1155/2016/6979435

**Published:** 2016-06-16

**Authors:** Dagmar Bruenig, Janine Lurie, Charles P. Morris, Wendy Harvey, Bruce Lawford, Ross McD Young, Joanne Voisey

**Affiliations:** ^1^School of Biomedical Sciences, Institute of Health and Biomedical Innovation (IHBI), 60 Musk Avenue, Queensland University of Technology, Kelvin Grove, QLD 4059, Australia; ^2^Gallipoli Medical Research Foundation, Greenslopes Private Hospital, Newdegate Street, Greenslopes, QLD 4120, Australia

## Abstract

Posttraumatic stress disorder (PTSD) is a debilitating condition that develops in some people after exposure to a traumatic event. Brain-derived neurotrophic factor (*BDNF*) is highly expressed in the mammalian brain and is thought to be involved in learning and memory processes. A nonsynonymous polymorphism in the* BDNF* gene, rs6265 (Val66Met), has been hypothesised to be associated with PTSD. Association studies examining the Val66Met polymorphism and PTSD have been inconclusive, likely due to the variability in type of trauma exposure analysed. Vietnam veterans (*n* = 257) screened for PTSD and controlled for trauma exposure were genotyped for* BDNF* Val66Met. The association was not significant so we incorporated our data into a meta-analysis to obtain greater statistical power. A comprehensive search of more than 1237 articles revealed eight additional studies suitable for meta-analysis (*n* = 3625). A random-effects meta-analysis observed a potential protective factor of the Val/Val genotype. After removing two studies with violation of Hardy-Weinberg equilibrium, findings for the Val/Val genotype reached significance. Subgroup analyses confirmed a trend for this finding. Limitations of some studies that inform this meta-analysis include poorly screened controls and a lack of examination of population stratification. Effectively designed studies should inform this line of research in the future.

## 1. Introduction

Posttraumatic stress disorder (PTSD) is a debilitating condition following from the experience of a severe traumatic event [1]. PTSD onset can be close to the traumatic event or delayed, and a majority of the population will never present with PTSD despite similar traumatic exposure [2]. Why some people develop clinical symptoms of PTSD while others do not is still unknown.

Memory processes such as recurring fearful memories and nightmares are central to PTSD symptomatology as they underpin the establishment of exacerbated fear responses (e.g., hypervigilance and startle, reviewed in [3]). The inability to extinguish fear response is thought to be fundamental to the persistence of the disorder [4], increasing anxiety states and perpetuating stress. Research on animal models and genetic predisposition in humans has identified a potential vulnerability to the establishment of fear memories and difficulties to subsequently extinguishing them, for example, Felmingham et al., 2013 [5].

Investigations of biological factors commonly associated with learning and memory formation have indicated that brain-derived neurotrophic factor (BDNF) may be a promising candidate. BDNF is a neurotrophin mediating synaptic plasticity [6]. It is highly expressed in the mammalian brain, especially in the hippocampus, which is functionally associated with learning and memory processes, reviewed in Yamada and Nabeshima [7]. Its binding to TrkB (tyrosine receptor kinase) causes intracellular cascades affecting neuronal development, plasticity, long-term potentiation, and apoptosis [8, 9]. The polymorphism rs6265, also known as Val66Met, of* BDNF* has been hypothesised to be important in fear learning and has shown some promising associations in animal models, reviewed in Andero and Ressler, 2012 [3]. The Met/Met genotype is associated with increased PTSD susceptibility, compromised memory performance, and reduced gene expression [10]. Reduced gene expression has been associated with reduced hippocampal volumes in animal models [10] and humans with PTSD [11].

Due to these findings and the role of fear memories in the development and maintenance of PTSD, research with human participants has attempted to identify associations between* BDNF* Val66Met and PTSD, but findings have been inconsistent. An intensive literature search across several databases with no language restriction yielded eight association studies of PTSD and Val66Met. Seven out of the eight studies did not find a significant association between the Met/Met genotype and PTSD. In the broader literature for Val66Met and disorders with high overlap with PTSD (e.g., depression and anxiety disorders), findings are equally mixed, for example, [12–14]. A meta-analysis of* BDNF* Val66Met and association with PTSD did not find an association overall but only in subgroup analyses for trauma exposed controls [15]. However, the researchers classified the study by Zhang et al. (2006) [16] as mixed race even though the study reported the population as European American. The authors also noted that the limited amount of studies reduced statistical power to detect an effect due to the relative rarity of the Met allele [15]. In summary, research into Val66Met and PTSD is inconclusive.

A case-control study was performed with participants who were thoroughly screened for trauma exposure and ethnicity and explored the role of* BDNF* Val66Met in PTSD. The results were added to a meta-analysis to expand on the findings from a previous meta-analysis of Val66Met and PTSD [15] in order to achieve higher statistical power to detect an effect. Based on previous studies in animal models [3] and with human participants, the hypothesis is that individuals carrying the Met allele will be at a higher risk of PTSD than those with the Val allele [10].

## 2. Methods

### 2.1. Case-Control Study

#### 2.1.1. Participants

For the case-control study, participants (*n* = 299) were sourced through a veterans hospital and the Returned and Services League of Australia with the aim of recruiting approximately equal group sizes for cases and controls. Inclusion requirements were deployment to Vietnam and being of Caucasian ethnicity; the only exclusion criterion was absence of trauma exposure (*n* = 32). As a pair of identical twins had participated, the information for one of the twins was not included. All included participants were male (*n* = 265). All participants were evaluated through semistructured interviews by trained psychiatrists at the hospital. A total of 158 participants were diagnosed with PTSD, with 107 with trauma exposure but no PTSD symptoms. Further nine participants were excluded due to ethnicity (*n* = 3) and problems with genotype calls (*n* = 6) resulting in a final cohort of *n* = 257 (PTSD: *n* = 151; no PTSD: *n* = 106) for chi-square analysis.

#### 2.1.2. Scales

Severity of PTSD was assessed by trained psychologists with the Clinician Administered PTSD Scale for DSM V [17], the gold standard procedure for PTSD assessment. Comorbidities were assessed using the Mini International Neuropsychiatric Interview (MINI), an instrument designed to assess major Axis 1 disorders in the DSM IV with high validity and reliability [18, 19].

#### 2.1.3. Genotyping

Blood samples were sent to the Australian Genome Research Facility (AGRF) for DNA extraction and genotyping. Upon arrival, samples were stored at −20°C. Genomic DNA was extracted from a 2 mL blood sample, using MACHEREY-NAGEL NucleoSpin L (MACHEREY-NAGEL GmbH & Co. KG, Düren, NRW, Germany). Quality of the DNA was assessed through resolution on a 0.8% agarose gel at 130 V for 60 minutes. Samples were normalised to 200 ng of DNA in 4 *μ*L.

Genotyping was performed using the* Illumina PsychArray-24 BeadChip* scanned with the Illumina iScan systems. Data analysis was performed using GenomeStudio v2011.1 (Illumina, San Diego, CA, USA) with Genotyping Module 1.9.4 software. Default settings by Illumina, the InfiniumPsychArray-24v1-1_A1 manifest and Infinium PsychArray-24v1-1_A1_ClusterFile cluster files, were applied. Across the entire array eight samples fell below the SNP call rate of 99% with the lowest call rate being 87% (range: 87% to 98%). Eleven samples were performed in duplicate for quality assurance with 100% reproducibility rate for Val66Met.

#### 2.1.4. Statistical Analysis

WinPEPI computer program for epidemiologists was used to calculate chi-squares, odds ratios, and 95% confidence intervals [20]. Hardy-Weinberg equilibrium (HWE) statistics were calculated using the Utility Programs for Analysis of Genetic Linkage [21]. All other analyses were conducted using SPSS Statistics Package 23 for Windows computers.

### 2.2. Meta-Analysis

#### 2.2.1. Literature Searches

Literature searches were conducted using the PubMed, PsycINFO, and PILOT databases to February 2016. No limitation for type of publication, language, or publication timeframe was set. Searches using the terms “PTSD,” “BDNF,” “rs6265,” and “Val66Met” in combination and separately were conducted and cross-referenced. Additional Google scholar alerts were set for the keywords PTSD and BDNF but did not yield further eligible studies. In addition, authors of relevant studies and known researchers in the field were contacted for potential additional information or unpublished data. One author group [22] provided additional information as participant recruitment had continued after their study results were published. The additional information was incorporated into the dataset of the originally published study [22] and the updated data were included in the current meta-analysis. The inclusion of additional data in the study did not change the overall findings of the original study. Another author group had reported on the role of* BDNF* Val66Met in a longitudinal study about the Wenchuan earthquake and the trajectory of PTSD symptom severity over the course of 18 months [23]. Upon request the authors kindly sent the genotype information for the cases and controls that had not been reported in the published paper. Lastly, our own data from the current case-control study were integrated in the meta-analysis.

Beyond the database searches, reference lists of all articles obtained were perused to identify additional studies. Articles were cross-referenced to ensure that independence of datasets was maintained; that is, publication duplication of the same data was controlled for.

In order to be included, studies had to be performed with human participants and had to use a case-control research design for associations with PTSD. Eight studies for PTSD and our own data met the inclusion criteria for analysis.  Table 1 lists all selected studies, their basic characteristics, genotype count, and HWE calculations.

#### 2.2.2. Statistical Analysis

Microsoft Excel was used to calculate individual odds ratios, log ratios, log standard errors, and 95% confidence intervals for each of the studies based on the formulae previously published [24]. Because not all studies reported HWE and one study had additional participants not previously reported, HWE statistics were calculated for all studies using the Utility Programs for Analysis of Genetic Linkage [21]. *Q* homogeneity tests were conducted to determine heterogeneity of effect size estimates [25]. Heterogeneity varied widely depending on inclusion or exclusion of studies in the main analyses and subanalyses. Hence, both fixed-effects and random-effects models were applied to calculate the combined odds ratio per genotype and on an allele basis as previously published [24], and subgroup analyses were performed to achieve refined data. The following analyses were pursued: for genotype analysis Val/Val genotype was compared to Val/Met and Met/Met. We also performed dominant and recessive analyses (Met/Met against pooled Val/Val and Val/Met and Val/Val against pooled Met/Met and Val/Met, resp.) both for individual studies and the combined meta-analysis. Furthermore, analyses on an allele level were performed, contrasting the risk allele (Met) against the Val allele. While the emphasis of this paper lies with the findings from the random-effects model due to its more conservative nature, findings from the fixed-effects model are presented, where heterogeneity was low-to-moderate. All data from the fixed-effects model (Table S1 and Table S2) can be found in the Supplementary Material available online at http://dx.doi.org/10.1155/2016/6979435. Fail Safe *N* was calculated [26]. This statistic indicates stability of the result under the assumption that study results may have been missed from the meta-analysis. Fail Safe *N* estimates the number of contradictory results needed to reverse the statistical significance of the meta-analytic effect size.

Due to the relatively small number of studies in this meta-analysis and given that the majority of the studies reported nonsignificant findings, publication bias could not be observed. Hence, further investigations of publication bias through funnel plots were not pursued.

## 3. Results

### 3.1. Case-Control Study

Table 2 shows an overview of the study cohort demographics and comorbidities by diagnosis.

An independent *t*-test revealed nonsignificant differences for age (*t*(265) = 1.274; *p* = 0.204) but, as would be expected, there were significant differences in CAPS severity scores between the PTSD and the no-PTSD group: *t*(262) = −13.348; *p* = 0.000. As expected, the distribution of the CAPS severity scores across the entire cohort was not normal and a nonparametric test (Kruskal-Wallis) was performed to identify potential differences between genotypes and CAPS severity. The test revealed a nonsignificant result: *p* = 0.345. To account for potential genotype associations within the PTSD group with regard to symptom severity, we performed an ANOVA with the patient cohort only, but the result was also nonsignificant: *F*(2) = 0.501; *p* = 0.607.

The PTSD and no-PTSD groups were both in HWE. The contingency table (Table 3) shows that all observations were above 5. The results for the chi-square test were not significant (*χ* ^2^(2) = 1.225; *p* = 0.54). Analysis based on allele frequency was also nonsignificant.

### 3.2. Meta-Analysis

Table 4 lists the individual studies with their respective converted odds ratios and confidence intervals for analysis of Val/Val genotype against heterozygous genotype and Met/Met genotype, respectively. Overall, the meta-analysis included 3625 participants, of which 1066 were cases and 2559 were controls. Of the 2559 controls, 1379 were trauma-exposed but had not developed symptoms of PTSD. The remaining 1180 control subjects were unscreened for trauma exposure. As had been reported by the respective authors and has been found with our own data, eight studies show nonsignificant results for an association of Val66Met with PTSD across the three genotypes. The only study showing significant results is the study by Zhang et al. [27]. However, HWE calculations in this study indicated that the control group was not in HWE (*p* = 0.005). We also ran all studies through dominant, recessive, and allelic analyses (Table 5). In these analyses, the same study [27] yielded significant results in the dominant and allele analyses. A further study was approaching significance in the dominant and allele analyses as well [23]. For this study, Hardy-Weinberg equilibrium was maintained. The subgroup analysis in Valente et al. [28] for trauma-exposed individuals also revealed a significant result but should be interpreted with caution due to the very low number of controls (*n* = 34) and the fact that this result could not be confirmed by analysis on an allele level. Similarly, we observed a significant effect in another study [29] for the dominant analyses but again this result was not confirmed by allelic analysis and the patient cohort was not in HWE.

For the overall meta-analysis, *Q* homogeneity analysis revealed high heterogeneity when all studies were included (Table 6). The random-effects model yielded nonsignificant results (Table 6). Because of the previously reported issues with HWE in both Zhang et al. (2014) [27] and Dretsch et al. [29], the studies were removed in two steps. Initially, only the study with HWE problems in the control cohort [27] was removed. The random-effects model remained nonsignificant. When the study with HWE problems in the patient cohort was also removed, a significant result was observed for the Val/Val genotype against the heterozygous genotype (Table 6). Similarly, for the recessive and dominant models, results remained nonsignificant when only the study with HWE deviation in the control group was removed [27]. However, the recessive model almost reached significance (OR = 0.90; CI 95%  [0.81; 1.00]) after both studies with HWE problems were removed. The dominant model as well as the allelic model remained nonsignificant. Heterogeneity in the allelic model dropped to a moderate level when only Zhang et al. 2014 [27] was removed and also when both studies with HWE violations were removed, and so the fixed-effects model was consulted as well. In both cases the fixed-effects model for allele analysis almost reached significance (OR = 1.12; CI 95%  [0.97; 1.28] and OR = 1.10; CI 95%  [0.96; 1.27], resp.; see Table S1). Fail Safe *N* was calculated indicating that about one study was needed to overturn the results of the overall meta-analysis with and without the studies with HWE violation. Fail Safe *N* information can be found in Table 6.

Due to differences in the genotype frequencies across ethnicities [32], subgroup analyses were conducted. Three studies included Caucasian participants [16, 22] and our own data; three studies included Asian participants [23, 30, 31]. The other three remaining studies had different mixed ethnic groups and were excluded from this subgroup analysis. The analysis for Caucasian ethnicity showed that the recessive model was approaching significance (OR = 0.92; CI 95%  [0.84; 1.01]). Both the dominant and the allelic models had low-to-moderate heterogeneity and the fixed-effects model was pursued but no significant findings were observed. For the Asian group, analyses revealed nonsignificant results across all models in the random-effects meta-analysis. However, heterogeneity analysis revealed low heterogeneity of the recessive model (Val/Val: *Q* = 2.55, *p* < 0.05; *I* ^2^ = 21.58). The fixed-effects model was approaching significance (OR = 0.79; CI 95%  [0.57; 1.09]; Table S2). All results for the random-effects model on ethnicity can be found in Table 7.

Four studies [22, 27, 29], including our current case-control study, reported using a control group with trauma exposure but no PTSD diagnosis (*n* = 638, *n* = 461, *n* = 226, and *n* = 257, resp.). Valente et al. (2011) [28] employed two control groups, a smaller one with trauma exposure and no PTSD symptomatology (*n* = 34) and a larger one of unscreened controls (*n* = 733). Hence, only the trauma-exposed subgroup was used for this subanalysis. As the subgroup analyses for trauma-controlled cohorts contained two studies deviating from HWE, these studies were removed in two steps. The findings for the random-effects model remained nonsignificant across all analyses when all studies were included and when only Zhang et al. (2014) [27] was removed. We pursued the fixed-effects model in the recessive analysis in this step and heterogeneity was low-to-moderate (Val/Val: *Q* = 5.17, *p* < 0.05; *I* ^2^ = 42.00), and the fixed-effects model was approaching significance (OR = 0.85; CI 95%  [0.69; 1.06]). When both studies deviating from HWE were removed, analysis of Val/Val against heterozygous genotype was approaching significance (OR = 0.84; CI 95%  [0.66; 1.08]), a finding that was confirmed by the fixed-effects model (OR = 0.84; CI 95%  [0.67; 1.07]). The recessive analysis was nonsignificant in the random-effects model but revealed moderate heterogeneity and the fixed-effects model was approaching significance (OR = 0.85; CI 95%  [0.67; 1.06]; Table S2).

## 4. Discussion

A case-control study and meta-analysis were conducted to investigate the role of* BDNF* Val66Met in PTSD susceptibility. This polymorphism has been implicated in the susceptibility to PTSD due to its association with decreased brain-derived neurotrophic factor levels leading to altered memory formation and brain volume, for example, [33]. Reduced hippocampal volumes have been observed in patients diagnosed with PTSD [11].

Our case-control study provided a tightly screened cohort for analysis. However, our study did not reach significance likely due to the sample size and low Met/Met genotype frequencies. This is in line with other studies published in this area, for example, [22]. We also could not establish differences in PTSD symptom severity by genotype, probably also due to sample size restriction and an additional loss of power with the use of a nonparametric test to account for the violation of normality in score distribution. Group differences in symptom severity between those diagnosed with PTSD and those with trauma exposure but no PTSD diagnosis were significant as would be expected.

Our data were used in a meta-analysis in an attempt to increase statistical power and evaluate the involvement of Val66Met in PTSD. Based on literature on animal and human studies, individuals with the Met allele were hypothesised to be associated with a higher likelihood of PTSD diagnosis than those with the Val allele. The overall meta-analysis revealed high heterogeneity of data and did not reveal a significant association. However, findings differed when two studies were removed because of deviating from HWE [27, 29]. We observed a significant effect in the overall analysis for a decreased risk of PTSD for the Val/Val genotype compared to the heterozygous genotype, albeit with a small effect size. A trend for a significant effect of the recessive model was also observed in the recessive analysis indicating that the Val/Val genotype may have protective properties towards the development of PTSD.

This trend was also observed in our subgroup analysis for ethnicity with an approach to significance for the Caucasian cohort in a random-effects model as well as for the Asian cohort in the fixed-effects model. Similarly, we found a trend for protective properties of the Val/Val genotype in the analysis with trauma-exposed controls only.

The results of this meta-analysis alter some of the findings of a previous meta-analysis using a random-effects model [15]. We expanded findings from that study with the addition of *n* = 1071 participants including our own study, a study on a Han-Chinese population [23], a mixed ethnicity but trauma-controlled study [29], and additional data for Caucasian participants [22]. We also applied a fixed-effects model to show potential trends. Our results show a trend towards an association of* BDNF* Val66Met with PTSD with the Val/Val genotype potentially exhibiting a protective factor. The previously published study could only find an effect of the polymorphism in the subgroup analysis for trauma exposure indicating an increased risk for Met carriers [15]. Our findings with regard to the protective mechanism of the Val/Val genotype are probably due to the higher occurrence of the Val allele in the general population as opposed to the relatively rare Met allele (Val allele, 80%, and Met allele, 20%, in a European population; see NCBI database [34]). We reported the fixed-effects model in our Results only in cases where heterogeneity was moderate-to-low. However, examination of the fixed-effects model across all analyses reveals a much stronger trend towards an involvement of the polymorphism in PTSD.

Even though we were able to add substantially to the meta-analysis, our results need to be interpreted with caution. Fail Safe *N* revealed that a very small number of studies can overturn the observed results which is not surprising given the small amount of studies published in this area so far. Clearly characterised study cohorts would enhance research in this area. The significant differences in Met/Met genotype distribution between ethnicities [32] underline the importance of designing ethnically controlled studies. Research with other genes has shown that ethnicity can play an important role in associations studies with significant results restricted to one specific ethnic group. One study, for example, found significant results only for a non-Hispanic black cohort of war veterans as opposed to non-Hispanic white veterans for the 5-HTTLPR polymorphism of the serotonin transporter gene* SLC6A4* [35]. Trauma type has been shown to influence likelihood of PTSD diagnosis [2] and PTSD severity differentially by gender [36] with PTSD rates within military samples being generally high (for an extensive review see [37]) and hence needs to be controlled for in future studies. Because of the significance of memory formation in the development of PTSD, screened controls, ideally with the same or similar trauma exposure, are required to determine an effect of the polymorphism in PTSD. We could not perform a subanalysis based on gender as gender type was not available in all studies. Research has suggested higher susceptibility [38] and heritability [39] for PTSD in women and trauma type influences PTSD diagnosis and severity differentially in women [36]. Gender based study cohorts would further improve research in this field.

## 5. Conclusion

This meta-analysis showed a trend for the involvement of* BDNF* Val66Met in PTSD. The trend shows a potential protective factor of the Val/Val genotype and in a fixed-effects model a trend for PTSD risk in Met carriers. However, the systematic investigation of published studies so far revealed that research in this area would benefit greatly from more clearly designed studies in terms of ethnic and control group definitions. It will be important to further research on the involvement of Val66Met in PTSD due to its potential role in fear memory formation and its interactions with the stress cascade.

## Supplementary Material

Table S1. Fixed effects model: Results from the overall meta-analysis including all studies and removal of Zhang et al. 2014 [27] and Dretsch et al. 2016 [29].
Table S2. Fixed effects model: Results from the subgroup analyses with and without Zhang et al., 2014 [27] and Dretsch et al. 2016 [29].

## Figures and Tables

**Table 1:** Overview of included studies, their characteristics, and Hardy-Weinberg equilibrium.

Study	Sample source	Sample gender	Trauma type	Diagnostic tool	Sample size	Patients	Controls	Ethn.	Val/Val	Val/Met	Met/Met	HWE
Zhang et al., 2006 [16]	Hospital	Mixed	N/A	SCID DSM III-R, SADS-L (PTSD section)	346	96	250	Cauc.	PTSD: 69 Controls: 166	PTSD: 26 Controls: 74	PTSD: 1 Controls: 10	PTSD: *p* = 0.39 Controls: *p* = 0.63

Valente et al., 2011 [28]	Hospital	N/A	Urban viol.	CIDI, SCID, CAPS, BAI, BDI, ETI	832	65	767	Braz.	PTSD: 48 PTSD−: 29 Controls: 555	PTSD: 15 PTSD−: 5 Controls: 164	PTSD: 2 PTSD−: 0 Controls: 14	PTSD: *p* = 0.54 PTSD−: *p* = 0.64 Controls: *p* = 0.64

Lee et al., 2006 [30]	Health centres; random controls	Mixed	N/A	SCID DSM IV, Korean version	268	107	161	Korean	PTSD: 28 Controls: 48	PTSD: 57 Controls: 82	PTSD: 22 Controls: 31	PTSD: *p* = 0.48 Controls: *p* = 0.70

Pivac et al., 2012 [22] (add. inf.)	Hospital	Male	Combat	SCID DSM IV, PANSS, CAPS	638	373	265	Cauc.	PTSD: 235 PTSD−: 173	PTSD: 126 PTSD−: 86	PTSD: 12 PTSD−: 6	PTSD: *p* = 0.32 PTSD−: *p* = 0.21

Zhang et al., 2014 [27]	US Army Special Ops.	N/A	Combat	PCL for DSM IV, Life Events Checklist	461	42	419	Mixed	PTSD: 20 PTSD−: 294	PTSD: 16 PTSD−: 104	PTSD: 6 PTSD−: 21	PTSD: *p* = 0.35 PTSD−: *p* = 0.005

Lyoo et al., 2011 [31]	Daegu registry, local comm.	Mixed	Subway disaster	CAPS, Structured Clinical Interviews for DSM IV	66	30	36	South Korean	PTSD: 10 Controls: 14	PTSD: 15 Controls: 18	PTSD: 5 Controls: 4	PTSD: *p* = 0.88 Controls: *p* = 0.62

Our study	Hospital; RSL	Male	Combat	CAPS, Semi-structured Clinical Interviews for DSM V	257	151	106	Cauc.	PTSD: 99 PTSD−: 71	PTSD: 46 PTSD−: 28	PTSD: 6 PTSD−: 7	PTSD: *p* = 0.82 PTSD−: *p* = 0.10

Li et al., 2016 [23]	High school	N/A	Natural disaster	PCL-C (DSM IV)	531	161	370	Han Chin.	PTSD: 39 PTSD−: 109	PTSD: 80 PTSD−: 190	PTSD: 42 PTSD−: 71	PTSD: *p* = 0.94 PTSD−: *p* = 0.46

Dretsch et al., 2016 [29]	US Army	N/A	Combat	PCL-M	226	41	185	Mixed	PTSD: 28 PTSD−: 129	PTSD: 8 PTSD−: 49	PTSD: 5 PTSD−: 7	PTSD: *p* = 0.009 PTSD−: *p* = 0.41

*Note*. Ethn., ethnicity; HWE, Hardy-Weinberg equilibrium; N/A, not available; Cauc., Caucasian; Braz., Brazilian; Chin., Chinese; viol., violence; PTSD−, trauma exposed but no PTSD diagnosis; controls, no trauma exposure; add. inf., includes additional information; comm., community; RSL, Returned Services League; SCID, Structured Clinical Interview for DSM; DSM, Diagnostic and Statistical Manual for Mental Disorders; SADS-L, schedule for affective disorders and schizophrenia-lifetime version; CIDI, Composite International Diagnostic Interview; CAPS, Clinician-administered PTSD scale; BAI, Beck Anxiety Interview; BDI, Beck Depression Inventory; ETI, Early Trauma Inventory; PANSS, Positive and Negative Syndrome Scale; PCL, Posttraumatic Stress Disorder Checklist.

**Table 2:** Demographics and clinical summary.

	PTSD	No PTSD
Age (M, SD)	68.47 (4.18)	69.13 (4.14)

Education level (counts)		
Less than year 10	26	6
Year 10	29	15
Vocational	32	17
Year 11 or 12	34	27
University	36	44

Direct combat experience (counts)	Yes: 137; no: 21	Yes: 85; no: 24

CAPS mean scores (SD)	15.71 (9.78)	2.68 (3.71)

Comorbidities (counts)^1^		
Major depression	21	1
Dysthymia	19	3
Suicide risk	31	1
Agoraphobia	33	6
Social phobia	8	0
Alcohol dependence	22	5
Alcohol abuse	3	1
Substance dependence	1	0
Substance abuse	0	0
Generalised anxiety disorder	12	2

*Note*. M, mean; SD, standard deviation; ^1^all comorbidity counts as per Mini International Neuropsychiatric Interview (MINI) for DSM IV [18, 19]. Only a subset of all comorbidities was shown. Rare comorbidities with no current information or both groups = 0 were excluded from the table.

**Table 3:** Association of genotype frequencies of *BDNF* Val66Met for participants with and without PTSD.

Val66Met	Genotype counts (%)			*p* value	Allele count	
	Val/Val	Val/Met	Met/Met		Val	Met
PTSD	99 (65.62)	46 (30.5)	6 (4.0)	0.542^*∗*^	244	58
No PTSD	71 (67.0)	28 (26.4)	7 (6.6)	0.435^*∗∗*^	170	42
Odds ratio (*p* value)	1	1.178 (0.811)	0.615 (0.639)		0.96 CI 95% [0.62; 1.50]	1

*Note*. ^*∗*^ *p* value determined by Pearson's *χ* ^2^ test; ^*∗∗*^ *p* value determined by Mantel-Haenszel test for trend in a given direction.

**Table 4:** Individual studies with their respective odds ratios and 95% confidence intervals.

Study	Val66Met	Genotype counts			*p* value
		Val/Val	Val/Met	Met/Met	
Zhang et al., 2006 [16]	PTSD	69 (71.88)	26 (27.08)	1 (1.04)^#^	0.306^*∗*^
	No PTSD	166 (66.40)	74 (29.60)	10 (4.00)	0.097^*∗∗*^
	Odds ratio (*p* value)	1	0.845 (0.779)	0.241 (0.202)	

Lee et al., 2006 [30]	PTSD	28 (26.17)	57 (53.27)	22 (20.56)	0.809^*∗*^
	No PTSD	48 (29.81)	82 (50.93)	31 (19.25)	0.282^*∗∗*^
	Odds ratio (*p* value)	1	1.192 (0.797)	1.217 (0.834)	

Valente et al., 2011 [28]					
All controls	PTSD	48 (73.85)	15 (23.08)	2 (3.08)^#^	0.756^*∗*^
	No PTSD	584 (76.14)	169 (22.03)	14 (1.83)	0.284^*∗∗*^
	Odds ratio (*p* value)	1	1.080 (0.962)	1.738 (0.751)	
Controls only	PTSD	48 (73.85)	15 (23.08)	2 (3.08)^#^	0.800^*∗*^
	No PTSD	555 (75.72)	164 (22.37)	14 (1.91)	0.314^*∗∗*^
	Odds ratio (*p* value)	1	1.058 (0.980)	1.652 (0.788)	
PTSD− only	PTSD	48 (73.85)	15 (23.08)	2 (3.08)^#^	0.333^*∗*^
	No PTSD	29 (85.29)	5 (14.71)	0 (0.00)^#^	0.075^*∗∗*^
	Odds ratio (*p* value)	1	1.165 (0.482)	1.860 (0.316)	

Lyoo et al., 2011 [31]	PTSD	10 (33.33)	15 (50.00)	5 (16.67)	0.775^*∗*^
	No PTSD	14 (38.89)	18 (50.00)	4 (11.11)^#^	0.253^*∗∗*^
	Odds ratio (*p* value)	1	1.167 (0.950)	1.750 (0.725)	

Pivac et al., 2012 [22]	PTSD	235 (63.00)	126 (33.78)	12 (3.22)	0.702^*∗*^
	No PTSD	173 (65.28)	86 (32.45)	6 (2.26)	0.229^*∗∗*^
	Odds ratio (*p* value)	1	1.079 (0.884)	1.472 (0.687)	

Zhang et al., 2014 [27]	PTSD	20 (47.62)	16 (38.10)	6 (14.29)	0.004^*∗*^
	No PTSD	294 (70.17)	104 (24.82)	21 (5.01)	0.000^*∗∗*^
	Odds ratio (*p* value)	1	**2.262 (0.048)**	**4.200 (0.023)**	

Li et al., 2016 [23]	PTSD	39 (24.22)	80 (49.69)	42 (26.09)	0.159^*∗*^
	No PTSD	109 (29.46)	190 (51.35)	71 (19.19)	0.033^*∗∗*^
	Odds ratio (*p* value)	1	1.177 (0.725)	1.653 (0.120)	

Dretsch et al., 2016 [29]	PTSD	28 (68.29)	8 (69.73)	5 (12.20)	0.077^*∗*^
	No PTSD	129 (69.73)	49 (26.49)	7 (3.78)	0.163^*∗∗*^
	Odds ratio (*p* value)	1	0.752 (0.755)	3.291 (0.129)	

Our study	PTSD	99 (65.62)	46 (30.5)	6 (4.0)	0.542^*∗*^
	No PTSD	71 (67.0)	28 (26.4)	7 (6.6)	0.435^*∗∗*^
	Odds ratio (*p* value)	1	1.178 (0.811)	0.615 (0.639)	

*Note*. ^#^At least one cell count less than 5; ^*∗*^ *p* value determined by Pearson's *χ* ^2^ test; ^*∗∗*^ *p* value determined by Mantel-Haenszel test for trend in a given direction; significant findings are in bold.

**Table 5:** Individual studies, their odds ratios, and 95% confidence intervals for recessive and dominant models on a genotype basis and on an allele model.

Study	Model	Odds ratios	95% CI
Zhang et al., 2006 [16]	Recessive	1.30	0.77; 2.17
	Dominant	0.25	0.03; 2.00
	Allele	0.74	0.47; 1.17

Lee et al., 2006 [30]	Recessive	0.83	0.48; 1.44
	Dominant	1.09	0.59; 2.00
	Allele	1.10	0.78; 1.56

Valente et al., 2011 [28]			
All controls	Recessive	0.88	0.50; 1.57
	Dominant	1.71	0.38; 7.68
	Allele	1.15	0.69; 1.92
Controls only	Recessive	0.91	0.51; 1.61
	Dominant	1.63	0.36; 7.33
	Allele	1.13	0.68; 1.88
PTSD− only	Recessive	0.49	0.16; 1.46
	**Dominant**	**0.08**	**0.02**; **0.36**
	Allele	2.16	0.77; 6.05

Lyoo et al., 2011 [31]	Recessive	0.79	0.29; 2.16
	Dominant	1.60	0.53; 3.88
	Allele	1.26	0.63; 2.55

Pivac et al., 2012 [22]	Recessive	0.91	0.65; 1.29
	Dominant	1.43	0.53; 3.87
	Allele	1.11	0.84; 1.47

Zhang et al., 2014 [27]	Recessive	0.39	0.20; 0.73
	**Dominant**	**3.16**	**1.20**; **8.33**
	**Allele**	**2.37**	**1.46**; **3.86**

Li et al., 2016 [23]	Recessive	0.77	0.50; 1.17
	Dominant	1.49	0.96; 2.30^*∗∗*^
	Allele	1.28	0.98; 1.66^*∗∗*^

Dretsch et al., 2016 [29]	Recessive	0.94	0.45; 1.94
	**Dominant**	**3.53**	**1.06**; **11.75**
	Allele	1.37	0.76; 2.47

Our study	Recessive	0.94	0.55; 1.59
	Dominant	0.59	0.19; 1.79
	Allele	0.96	0.62; 1.50

*Note*. Significant findings are in bold; ^*∗∗*^approaching significance.

**Table 6:** Results of the overall meta-analysis including all studies and stepwise removal of Zhang et al. (2014) [27] and Dretsch et al. (2016) [29] in a random-effects model.

Analysis	Model	Odds ratio	95% CI	*I* ^2^	Fail safe *N*
All studies	Val/Val versus Val/Met	0.92	0.57; 1.47	82.87	N/A
	Val/Val versus Met/Met	0.87	0.15; 4.97	96.28	N/A
	Recessive	0.87	0.56; 1.34	89.35	N/A
	Dominant	1.44	0.61; 3.38	86.99	N/A
	Allele	1.20	0.65; 2.20	95.02	1.20

Zhang et al. (2014) [27] removed	Val/Val versus Val/Met	0.97	0.70; 1.36	62.25	N/A
	Val/Val versus Met/Met	1.02	0.19; 5.56	95.51	0.14
	Recessive	0.65	0.39; 1.10	N/A	N/A
	Dominant	1.24	0.60; 2.60	78.30	1.46
	Allele	1.10	0.88; 1.37	55.34	0.60

Zhang et al. (2014) [27] and Dretsch et al. (2016) [29] removed	Val/Val versus Val/Met	**0.82**	**0.70**; **0.96**	N/A	N/A
	Val/Val versus Met/Met	1.22	0.19; 7.66	95.79	1.10
	Recessive	0.90	0.81; 1.00^*∗∗*^	N/A	N/A
	Dominant	1.07	0.48; 2.39	81.40	0.37
	Allele	1.08	0.85; 1.35	58.51	0.38

*Note*. Significant findings are in bold; ^*∗∗*^approaching significance.

**Table 7:** Results from the subgroup analyses with and without Zhang et al. (2014) [27] and Dretsch et al. (2016) [29] from the random-effects model.

Analysis	Model	Odds ratio	95% CI	*I* ^2^	Fail Safe *N*
Ethnicity					
Caucasian	Val/Val versus Val/Met	1.14	0.84; 1.55	N/A	0.71
	Val/Val versus Met/Met	1.72	0.00; 652.97^*∗*^	98.46	3.60
	Recessive	0.92	0.84; 1.01^*∗∗*^	N/A	N/A
	Dominant	0.73	0.26; 2.09	48.90	N/A
	Allele	0.96	0.73; 1.27	35.96	N/A
Asian	Val/Val versus Val/Met	0.80	0.57; 1.11	N/A	N/A
	Val/Val versus Met/Met	0.67	0.20; 2.19	83.90	N/A
	Recessive	0.79	0.54; 1.16	21.58	N/A
	Dominant	1.34	0.53; 3.39	80.38	1.71
	Allele	1.20	0.70; 2.08	82.22	1.04

PTSD−	Val/Val versus Val/Met	0.86	0.40; 1.84	89.50	N/A
	Val/Val versus Met/Met	0.61	0.09; 4.00	95.33	N/A
	Recessive	0.70	0.30; 1.68	93.48	N/A
	Dominant	0.96	0.009; 10.65	97.09	N/A
	Allele	1.44	0.55; 3.78	96.91	2.64
PTSD− Zhang et al. (2014) [27] removed	Val/Val versus Val/Met	0.95	0.54; 1.68	69.17	N/A
	Val/Val versus Met/Met	0.66	0.18; 2.55	88.64	N/A
	Recessive	0.85	0.65; 1.10	22.69	N/A
	Dominant	0.71	0.04; 11.63	97.19	N/A
	Allele	1.24	0.83; 1.83	76.44	1.41
PTSD− Zhang et al. (2014) [27] and Dretsch et al. (2016) [29] removed	Val/Val versus Val/Met	0.84	0.66; 1.08^*∗∗*^	6.22	N/A
	Val/Val versus Met/Met	0.85	0.26; 2.80	84.52	N/A
	Recessive	0.82	0.60; 1.15	42.00	N/A
	Dominant	0.57	0.03; 11.16	97.59	N/A
	Allele	1.21	0.77; 1.91	80.97	1.06

*Note*. ^*∗∗*^Approaching significance; ^*∗*^very wide confidence intervals potentially due to frequencies.
