# Supplementary material for: A Case-Control Study and Meta-Analysis Reveal BDNF Val66Met Is a Possible Risk Factor for PTSD
Source: Neural Plast. 2016 Jun 16;2016:6979435. doi: 10.1155/2016/6979435 (PMC4928001; doi:10.1155/2016/6979435)
Supplement: Supplementary file 1 — Table S1. Fixed effects model: Results from the overall meta-analysis including all studies and removal of Zhang et al. 2014 [27] and Dretsch et al. 2016 [29]. Table S2. Fixed effects model: Results from the subgroup analyses with and without Zhang et al., 2014 [27] and Dretsch et al. 2016 [29]. [file 6979435.f1.docx]

**Supplementary Material**

**A case-control study and meta-analysis reveal *BDNF* Val66Met is a possible risk factor for PTSD**

The following tables show the results for the fixed effects model meta-analyses.

The data is only discussed in text if calculations showed low to moderate heterogeneity.

As can be seen from the tables below, the fixed effects model yields more significant findings and findings that are approaching significance towards a support of the hypothesis. Due to various degrees of observed heterogeneity and the more conservative approach of the random effects model, the results here are for comparison purposes.

Table S1. Fixed effects model: Results from the overall meta-analysis including all studies and removal of Zhang et al. 2014 [27] and Dretsch et al. 2016 [29]

| **Analysis** | **Model** | **Odds Ratio** | **95% CI** | ***I^2^*** | **Fail Safe *N*** |
| --- | --- | --- | --- | --- | --- |
| All studies | Val/Val vs Val/Met | 0.86 | 0.74; 1.06** | 82.87 | N/A |
|  | Val/Val vs Met/Met | **0.67** | **0.49; 0.92** | 96.28 | N/A |
|  | Recessive | 0.85 | 0.72; 1.01** | 89.35 | N/A |
|  | Dominant | **1.42** | **1.08; 1.89** | 86.99 | 2.55 |
|  | Allele | **1.18** | **1.03; 1.34** | 95.01 | 1.08 |
| Zhang et al., 2014; removed | Val/Val vs Val/Met | 0.93 | 0.77; 1.13 | 62.24 | N/A |
|  | Val/Val vs Met/Met | 0.75 | 0.54; 1.05** | 95.51 | N/A |
|  | Recessive | 0.90 | 0.76; 1.09** | N/A | N/A |
|  | Dominant | 1.32 | 0.99; 1.78** | 78.30 | 1.95 |
|  | Allele | 1.12 | 0.97; 1.28** | 55.36 | 0.70 |
| Zhang et al., 2014 and Dretsch et al. 2016 removed | Val/Val vs Val/Met | 0.90 | 0.75; 1.09** | N/A | N/A |
|  | Val/Val vs Met/Met | 0.81 | 0.57; 1.14 | 95.79 | N/A |
|  | Recessive | 0.91 | 0.76; 1.09** | N/A | N/A |
|  | Dominant | 1.32 | 0.99; 1.78** | 81.40 | 1.32 |
|  | Allele | 1.10 | 0.96;1.27** | 58.51 | 0.52 |

*Note*: Significant findings in bold; ** approaching significance

Table S2. Fixed effects model: Results from the subgroup analyses with and without Zhang et al., 2014 [27] and Dretsch et al. 2016 [29]

| **Analysis** | **Model** | **Odds Ratio** | **95% CI** | ***I^2^*** | **Fail Safe *N*** |
| --- | --- | --- | --- | --- | --- |
| Ethnicity | Val/Val vs Val/Met | 0.96 | 0.75; 1.24 | N/A | N/A |
| *Caucasian* | Val/Val vs Met/Met | 1.52 | 0.76; 3.07 | 98.46 | 2.6 |
|  | Recessive | 0.99 | 0.77; 1.26 | N/A | N/A |
|  | Dominant | 0.83 | 0.41; 1.67 | 48.91 | N/A |
|  | Allele | 0.98 | 0.80; 1.22 | 35.96 | N/A |
| *Asian* | Val/Val vs Val/Met | 0.80 | 0.57; 1.12 | N/A | N/A |
|  | Val/Val vs Met/Met | 0.67 | 0.44; 1.00** | 83.90 | N/A |
|  | Recessive | 0.79 | 0.57; 1.09** | 21.58 | N/A |
|  | Dominant | 1.35 | 0.96; 1.91** | 80.38 | 1.75 |
|  | Allele | 1.21 | 0.99; 1.48** | 82.22 | 1.07 |
| PTSD- | Val/Val vs Val/Met | 0.83 | 0.66; 1.03** | 89.50 | N/A |
|  | Val/Val vs Met/Met | **0.56** | **0.39; 0.82** | 95.33 | N/A |
|  | Recessive | **0.79** | **0.64; 0.97** | 93.48 | N/A |
|  | Dominant | 1.39 | 1.00; 1.93** | 97.09 | 1.17 |
|  | Allele | **1.28** | **1.09; 1.50** | 96.91 | 1.68 |
| PTSD-  Zhang et al. 2014 removed | Val/Val vs Val/Met | 0.89 | 0.70; 1.12 | 69.17 | N/A |
|  | Val/Val vs Met/Met | **0.65** | **0.43; 0.97** | 88.64 | N/A |
|  | Recessive | 0.85 | 0.69; 1.06** | 22.69 | N./A |
|  | Dominant | 1.25 | 0.88; 1.77 | 97.19 | 0.74 |
|  | Allele | **1.19** | **1.01; 1.41** | 76.44 | 1.14 |
| PTSD-  Zhang et al. 2014 and Dretsch et al. 2016 removed | Val/Val vs Val/Met | 0.84 | 0.67; 1.07** | 6.22 | N/A |
|  | Val/Val vs Met/Met | 0.71 | 0.46; 1.10 | 84.52 | N/A |
|  | Recessive | 0.85 | 0.67; 1.06** | 42.00 | N/A |
|  | Dominant | 1.13 | 0.79; 1.63 | 97.59 | 0.40 |
|  | Allele | 1.17 | 0.99; 1.40** | 80.97 | 0.88 |

*Note*: Significant findings in bold; ** approaching significance; *very wide confidence intervals potentially due to frequencies
